# Functional genomics approaches to improve pre‐clinical drug screening and biomarker discovery

**DOI:** 10.15252/emmm.202013189

**Published:** 2021-07-13

**Authors:** Long V Nguyen, Carlos Caldas

**Affiliations:** ^1^ Department of Oncology and Cancer Research UK Cambridge Institute Li Ka Shing Centre University of Cambridge Cambridge UK; ^2^ Cancer Research UK Cambridge Cancer Centre Cambridge UK

**Keywords:** biomarker discovery, cancer models, drug screening, pharmacogenomics, single‐cell sequencing, Cancer, Chromatin, Epigenetics, Genomics & Functional Genomics

## Abstract

Advances in sequencing technology have enabled the genomic and transcriptomic characterization of human malignancies with unprecedented detail. However, this wealth of information has been slow to translate into clinically meaningful outcomes. Different models to study human cancers have been established and extensively characterized. Using these models, functional genomic screens and pre‐clinical drug screening platforms have identified genetic dependencies that can be exploited with drug therapy. These genetic dependencies can also be used as biomarkers to predict response to treatment. For many cancers, the identification of such biomarkers remains elusive. In this review, we discuss the development and characterization of models used to study human cancers, RNA interference and CRISPR screens to identify genetic dependencies, large‐scale pharmacogenomics studies and drug screening approaches to improve pre‐clinical drug screening and biomarker discovery.

## Graphical abstract

This Review provides an overview of currently used cancer models and genomics approaches to uncover genetic dependencies, identify novel therapeutic targets, and discover biomarkers that can predict response to drug therapy.

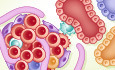

**Glossary**
Genotype–phenotype relationships: The association between a specific genotype (i.e., genetic alteration) and the resulting phenotype (i.e., observable characteristic). In cancer biology, this refers to how specific genetic alterations produce changes in the properties of tumour cells.
Patient avatars: Patient‐derived tumour xenografts that are co‐treated with the same therapies as the patient to mimic changes that would occur in the patient in order to evaluate response to therapy and changes in clonal heterogeneity.
Predictive biomarker: A marker (e.g., genetic abnormality or protein) that can be used to predict the response of a tumour to a specific therapy.
Spatiotemporal heterogeneity: This refers to the different clones (that harbour distinct genomic aberrations) present in different areas of a tumour and that appeared at different time points in disease progression.
Targeted therapy: Drug therapy directed at a specific gene product or pathway, as opposed to chemotherapy, which is less specific and targets all proliferating cells.
Tumour microenvironment: Interactions between tumour cells and the cells and growth factors within its surrounding environment, such as stromal cells, blood vessels and infiltrating inflammatory cells.

## Introduction

Rapidly developing sequencing technology, in conjunction with advances in computational analysis, has culminated in the genomic and transcriptomic characterization of individual patients’ tumours, bringing the possibility of precision‐medicine to the clinic. However, a remaining challenge is how to use this information to identify therapeutic targets and predictive biomarkers to guide clinical decision‐making about whether a particular drug is likely to be effective for the treatment of a specific patient’s disease.

There have been a number of notable successes in the development of targeted therapy. Some of these include the use of tyrosine kinase inhibitors such as Imatinib for chronic myelogenous leukaemia harbouring the BCR‐ABL translocation (Annunziata *et al*, 2020), BRAF/MEK inhibitors for BRAF mutated cancers (Zaman *et al*, 2019), HER2‐targeted therapies in breast cancer (Wang & Xu, 2019), and inhibitors of EGFR or ALK kinases in lung adenocarcinomas driven by EGFR mutation or ALK fusions (Bernicker *et al*, 2019). In many other cancer types, discoveries of predictive biomarkers for clinical use have remained elusive. There are a number of reasons for this. The detection of a specific mutation does not necessarily mean it is a driver mutation, the mutation may not be present in a majority of the cells that drive disease progression, and rapid development of resistance may occur from the selection or *de novo* generation of drug‐resistant clones. To overcome these challenges, efforts have been taken to model cancer in different systems, study how specific genomic alterations result in changes to tumour growth, and test different therapies in these model systems as a surrogate readout for how the patient’s cancer will respond to treatment. These approaches offer the opportunity to uncover genetic dependencies, identify novel therapeutic targets and define mechanisms of drug resistance.

In this review, we will discuss the advantages and limitations of different models of human cancer, including human cancer cell lines, patient‐derived tumour organoids (PDTOs), and patient‐derived tumour xenografts (PDTXs). We will also review RNA interference (RNAi) and CRISPR technologies applied to functional genomic screens for the discovery of novel therapeutic targets, drug screens, and combination therapy screens using different cancer models in order to identify novel molecular biomarkers that can predict response to drug therapy.

## Models to study human cancers

### Human cancer cell lines

Hundreds of human cancer cell lines have been established and are the most widely used to study cancer biology and drug screening. Resources with comprehensive genomic information about these cell lines include the Cancer Cell Line Encyclopedia (CCLE; Barretina *et al*, 2012), the Genomics of Drug Sensitivity in Cancer (GDSC; Yang *et al*, 2013; Iorio *et al*, 2016), the Cell Model Passports (van der Meer *et al*, 2019), the Cellosaurus (Bairoch, 2018), and the COSMIC catalogue of somatic mutations (Tate *et al*, 2019). There are also gene expression, single nucleotide polymorphism, gene fusion (Klijn *et al*, 2015) and proteomic data on these cell lines, from the MD Anderson Cell Lines Project (MCLP) (Li *et al*, 2017) and the NCI‐60 cell lines (Nishizuka *et al*, 2003; Gholami *et al*, 2013).

**Figure 1:**
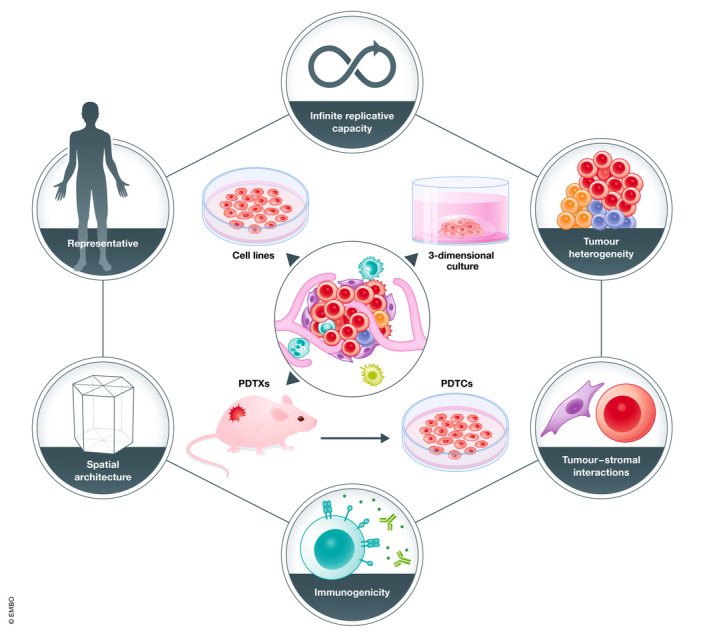
Models to study human cancers The main models to study human cancers are human cancer cell lines, 3‐dimensional *in vitro* culture systems such as those used to grow PDTOs, PDTXs and PDTCs as depicted in the diagram above. PDTCs are short‐term *ex vivo* cultures of cells dissociated from PDTXs and can be used for drug screening. The outer circles represent different characteristics of model systems that may be required to study different aspects of cancer biology. For example, human cancer cell lines have been immortalized and thus have infinite replicative capacity *in vitro* (Sachs & Clevers, 2014). PDTOs are more representative of the full spectrum of human cancers, given the high rate at which they can be established (Sachs *et al*, 2018). Both PDTOs and PDTXs retain spatial architecture (Clevers, 2016; McGranahan & Swanton, 2017), PDTXs retain tumour‐stromal interactions important for studying the role of the microenvironment in tumour biology (Julien *et al*, 2012; Peng *et al*, 2013). Adaptations of PDTO models can be made to study tumour‐stromal interactions (Aboulkheyr Es *et al*, 2018), and interactions between the tumour and immune cells (Neal *et al*, 2018), as opposed to PDTXs which are grown in mice typically lacking a functional immune system (Shultz *et al*, 2014; Byrne *et al*, 2017).

The main advantage of human cancer cell lines is that they can be easily maintained *in vitro* through serial passaging and expansion, thus representing an unlimited resource (Fig 1). Cell lines are generally perceived to be homogeneous, offering a simplistic model for studying the functional consequences of different genomic perturbations, making them ideal for high‐throughput drug screening and functional assays (Sachs & Clevers, 2014). The lack of phenotypic and genetic heterogeneity (compared to the original tumour) can be attributed to the high selection pressures in early passages when the cells are forced to grow in 2D culture. There may also be genetic deviation over time whereby the cell line is no longer representative of the original patient’s disease (Stein *et al*, 2004). This is demonstrated when gene expression profiles of tumours more closely resemble normal tissues than cancer cell lines (van Staveren *et al*, 2009). However, recent whole‐exome sequencing of 106 human cancer cell lines showed they are in fact highly heterogeneous. This was attributed to the presence of pre‐existing subclones and the emergence of new genetic variants, which contribute to genetic instability and may ultimately affect drug sensitivity screens (Ben‐David *et al*, 2018).

Human cancer cell lines are also difficult to establish from primary patient material. Faster growing cancer cells are more likely to adapt to *in vitro* growth conditions compared to slow‐growing cells, thus resulting in an over‐representation of advanced/metastatic cancers as cell lines (Masters, 2000). Another limitation of human cancer cell lines is that there have been significant differences found in their transcriptomic profiles compared to primary human tumour samples (Najgebauer *et al*, 2020). These differences include an upregulation of cell‐cycle‐related pathways and downregulation of immune pathways in cell lines compared to primary tumours (Yu *et al*, 2019). The other drawback of cell lines is the lack of tumour architecture and microenvironment, which may be a reason why drug therapies identified from work with cell lines do not always translate into clinical efficacy. Nevertheless, human cancer cell lines are still widely used to model disease and test drug sensitivity due to their ease for incorporation into high‐throughput screens and also hold promise for unveiling novel drug targets by repurposing already known drug therapies (Pushpakom *et al*, 2019).

### Patient‐derived tumour organoids

Organoids were initially established from normal tissues, generated from either pluripotent stem cells, or adult organ‐restricted stem cells, and self‐organize into 3D structures that contain the same diversity of cell types and architecture of the original organ tissue (Clevers, 2016). Recently, organoids have also been generated from numerous human tumours, including breast, prostate, lung, colorectal, renal, bladder, pancreatic, oesophageal, gastric, liver, and ovarian cancers (Bleijs *et al*, 2019).

The main advantage of PDTOs is that they appear to regenerate the heterogeneity seen in human cancers (Fig 1). For example, colorectal PDTOs derived from single cells have demonstrated extensive mutational diversification and a differential response to anti‐cancer drugs (Roerink *et al*, 2018). Weeber and colleagues also showed that colorectal PDTOs retain 90% of somatic mutations and DNA copy number alterations (CNAs) compared to original biopsy specimens (Weeber *et al*, 2015).

Modelling the tumour microenvironment is crucial to investigating the anti‐tumour effects of immune checkpoint inhibitors. However, similar to cell lines, PDTO culture systems do not preserve the tumour microenvironment. To circumvent this limitation, several groups have developed microfluidics platforms to co‐culture PDTOs with other cell types, such as adipocytes, lymphocytes, macrophages and myofibroblasts, in an attempt to simulate the interactions between the tumour and cellular components of the microenvironment (Aboulkheyr Es *et al*, 2018). Alternatively, Neal and colleagues cultured PDTOs with an air–liquid interface to preserve stromal architecture and functional tumour‐infiltrating lymphocytes. They validate this system on colorectal, pancreas, lung, biliary and primary CNS cancers and show that tumour‐infiltrating lymphocytes constituted the full T‐cell receptor spectrum of the original tumour, and further that treatment with anti‐PD‐1 and anti‐PD‐L1 immune checkpoint inhibitors resulted in the expected tumour cytotoxicity (Neal *et al*, 2018).

Organoid biobanks have been established in numerous tissue systems, including colorectal (van de Wetering *et al*, 2015), and breast (Sachs *et al*, 2018), some of which have been extensively characterized. For example, Sachs and colleagues established a biobank of human breast organoids with a > 80% success rate of PDTO formation and showed the organoids closely resemble patients’ tumours in terms of histopathology, hormone and HER2 receptor status, CNA and mutational profiles, and gene expression, which were preserved even after extended passaging *in vitro*. The ‘Cell Model Passports’ is a helpful resource that compiles clinical information and sequencing data from PDTOs derived from different tumour types (van der Meer *et al*, 2019).

### Patient‐derived tumour xenografts

In general, PDTXs are established by taking freshly obtained tumour cells from a patient and engrafting these cells into immunodeficient mice (Fig 1). While this model is thought to most closely resemble the original patient’s tumour, since cellular and genetic heterogeneity, tumour architecture and microenvironment are preserved, these models can be both time consuming and expensive. Generation of a PDTX model can require up to 4–8 months, limiting its usefulness in the clinic for real‐time testing of response to treatment (Hidalgo *et al*, 2014).

Tumour engraftment is largely dependent on the use of mice with deficient immune systems. Recently, highly immune‐deficient NSG mice, which lack mature T, B and natural killer cells, have allowed higher engraftment rates, as compared to earlier models of immune‐deficient mice, such as NOD‐SCID, and athymic mice (Shultz *et al*, 2014; Byrne *et al*, 2017). One concern in this situation is that the process whereby PDTXs engraft and propagate may not resemble human cancers that usually initiate in an immune‐competent host. Moreover, PDTXs in immune‐deficient hosts may represent a poor model to study the effects of immunotherapy on the tumour microenvironment. In PDTXs, the microenvironment is largely thought to be preserved on initial engraftment as human stromal components are also engrafted, though over time, human stromal cells are replaced by murine stromal cells (Julien *et al*, 2012; Peng *et al*, 2013). This can be associated with downregulation of genes corresponding to cell adhesion and immune response (Morgan *et al*, 2017). There may also be metabolic differences in PDTXs established orthotopically versus subcutaneously, a finding that was attributed to differences in the microenvironment (Zhan *et al*, 2017). Conversely, recent proteomic analyses demonstrated in a PDTX model of colorectal cancer that infiltrating murine stromal cells adopted metabolic profiles that became “human‐like” and recapitulated what was found in original patient tumours (Blomme *et al*, 2018).

Due to spatiotemporal heterogeneity observed in different tumours, establishing PDTXs from a single small sample of the original tumour will likely not capture the full mutational diversity present in a patient’s disease (McGranahan & Swanton, 2017). This has been illustrated in PDTX models of melanoma (Rabbie *et al*, 2020), where the spatial heterogeneity of different genetic clones results in variable responses to treatment (Kemper *et al*, 2015; Wellbrock, 2015). This suggests that spatial clonal heterogeneity can have a substantial impact on studies using PDTXs to study tumour biology and response to therapy.

Numerous efforts have been made to establish PDTXs as a resource to study tumour heterogeneity and responses to therapy (Bertotti *et al*, 2011; Byrne *et al*, 2017; Woo *et al*, 2021). These studies have demonstrated that PDTXs largely retain the same histologic and genetic characteristics of the original patient’s tumour (Hidalgo *et al*, 2014). This appears to be true in numerous tumour types, including breast (DeRose *et al*, 2011; Bruna *et al*, 2016a), lung (Fichtner *et al*, 2008), colon (Dalerba *et al*, 2011; Julien *et al*, 2012), prostate (Palanisamy *et al*, 2020), melanoma (Krepler *et al*, 2017), bladder (Kim *et al*, 2020) and pancreatic cancers (Loukopoulos *et al*, 2004), among others. However, PDTXs are also generated with higher efficiency from invasive and metastatic tumours compared to slow‐growing or non‐metastatic tumours. For example, triple‐negative breast cancers have shown a higher engraftment efficiency compared to hormone‐receptor‐positive breast cancers, that are generally thought to be less aggressive clinically (DeRose *et al*, 2011; Zhang *et al*, 2013). This has led to the establishment of PDTX biobanks that are over‐representative of more aggressive tumours.

One major concern has been that clonal selection pressures on initial engraftment and subsequent passaging of PDTXs may alter the clonal composition of the PDTXs, such that it does not entirely mimic the original patient tumour (Ben‐David *et al*, 2017). Eirew and colleagues analysed the clonal dynamics of breast tumour xeno‐engraftment into immunodeficient mice and found variable clonal selection pressures between samples (Eirew *et al*, 2015). However, an international consortium analysed the CNA profiles of over 500 PDTX models at high‐resolution and found a strong conservation of CNAs between patient tumours and late passage PDTXs (PDXNET Consortium *et al*, 2021). Together, these studies suggest that clonal selection can be variable between PDTX models, though in many cases are relatively conserved. Transcriptomic analyses of established PDTXs have also demonstrated PDTXs mirror the gene expression patterns observed in original patient tumours (Dalerba *et al*, 2011), and they appear to maintain clonal intra‐tumour heterogeneity and tumour architecture, even with serial passaging (Bruna *et al*, 2016a).

Furthermore, short‐term cultures can be generated from PDTXs, called PDTX‐derived tumour cells (PDTCs, Fig 1), and these can be used for pre‐clinical high‐throughput drug screens (Bruna *et al*, 2016b; Georgopoulou *et al*, 2021). While establishing PDTXs can be resource intensive, they most closely resemble the original patient tumour and microenvironment, and thus hold the most promise for validating targeted therapies that will have meaningful clinical outcomes.

## Identifying novel predictive biomarkers for clinical use

For the purposes of cancer treatment, an ideal predictive biomarker is one that can be easily assessed from a patient’s biopsy specimen and has a high likelihood of predicting response to a particular line of therapy. Tissue on which the biomarker can be assessed can be obtained by biopsy of the primary tumour or sites of metastases, or liquid biopsy in the form of circulating tumour cells or circulating cell‐free tumour DNA (ctDNA; Wan *et al*, 2017). ctDNA is released into the bloodstream from the turnover of tumour cells and has been used to monitor disease burden (Dawson *et al*, 2013), detect the presence of clinically actionable mutations, and to analyse clonal evolution in cancer (Murtaza *et al*, 2015). ctDNA thus has appeal for obtaining helpful biological information from non‐invasive liquid biopsy of a patient’s blood. The biomarker itself can be the presence or absence of genetic alterations such as mutations, translocations, copy number alterations, epigenetic modifications or gene expression profiles indicating dependency on a specific targetable pathway.

While there have been a number of disease context‐specific practice‐changing discoveries, such as HER2‐targeted therapy in HER2 amplified breast and gastroesophageal cancers, or poly(ADP‐ribose) polymerase (PARP) inhibition in ovarian cancers with homologous DNA repair deficiency, there still appear to be a range of responses to appropriately selected therapy and the eventual development of drug resistance. Hence, there is value in the discovery of predictive biomarkers not only for other disease‐specific contexts where there are no reliable biomarkers to guide the choice of appropriate therapy, but also for the prediction of drug resistance, and the selection of appropriate therapy once this has developed.

There are a number of approaches to discover novel biomarkers in cancer. These include genomic and transcriptomic profiling, proteomic approaches and metabolomic approaches, all of which are beyond the scope of our review but have been covered in detail elsewhere (Armitage & Barbas, 2014; Hristova & Chan, 2019). We will focus on the identification of novel therapeutic targets, in the context of genetic alterations that can predict response to specific drug therapies and thus act as useful predictive biomarkers.

## Functional genomic screens for discovery of novel therapeutic targets

**Figure 2:**
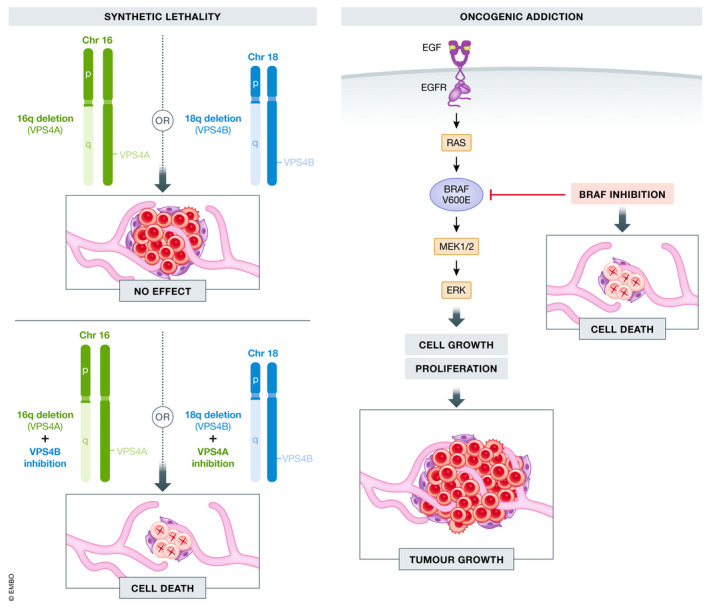
Depiction of genetic dependencies in cancer Synthetic lethality is depicted on the left, using VPS4A on chromosome 16q and VPS4B on chromosome 18q as an example. When 16q or 18q deletions occur separately, there is no effect on tumour growth. However, when VPS4B is inhibited in tumour cells with 16q deletion, or when VPS4A is inhibited in tumour cells with 18q deletion, synthetic lethality occurs and there is cell death (Neggers *et al*, 2020). Oncogenic addiction is depicted on the right, using BRAF as an example. In cells harbouring an oncogenic BRAF‐V600E mutation, there is constitutive activation of the signalling pathway leading to cell growth and proliferation. However, these cells are particularly sensitive to knockdown or inhibition of BRAF, which leads to cell death, as the cells are dependent on this signalling pathway for continued growth (Settleman, 2012).

The basic premise of a functional genomic screen is that disruption of a key gene product essential for tumour growth will result in tumour regression and death. This particular gene product thus represents a potential therapeutic target. If the gene of interest represents an essential pathway in normal cells as well, the therapeutic index is expected to be narrow, and treatment associated with high levels of toxicity. Therefore, identifying genetic dependencies such as oncogenic addiction, where the cancer cells are driven by and dependent on a certain oncogene, or synthetic lethality, where the acquisition of a second genetic alteration leads to cell death, may uncover more ideal therapeutic targets (Fig 2).

The concept of synthetic lethality was originally described in Drosophila as recessive lethality, in which the loss of one gene has little effect on cell viability, but the additional loss of a second gene leads to cell death. Extrapolated to the context of cancer, when the cancer cells harbour a specific fixed genetic alteration of one gene, the additional loss of a second gene either by mutation, deletion or pharmacological inhibition, will result in selective cell death of cancer cells sparing normal cells that do not harbour the specific fixed genetic alteration (Kaelin, 2005). The first most clinically relevant application of synthetic lethality is the use of PARP inhibitors in BRCA1‐ or BRCA2‐deficient tumours (Fong *et al*, 2009). PARP, BRCA1 and BRCA2 are components required for efficient DNA repair, and therefore, tumour cells that already harbour a BRCA1 or BRCA2 mutation will have increased sensitivity to PARP inhibitor therapy (Bryant *et al*, 2005; Farmer *et al*, 2005). Normal cells have at least one functioning copy of BRCA1 or BRCA2 and are thus largely spared, limiting toxicity from PARP inhibitor therapy. PARP inhibitors have demonstrated a progression‐free survival benefit as maintenance therapy for treatment‐naïve advanced/metastatic ovarian cancer in patients with a germline or somatic BRCA1 or BRCA2 mutation (Moore *et al*, 2018), and an overall survival benefit as maintenance therapy for similar patients who experience disease recurrence (Pujade‐Lauraine *et al*, 2017), and are now routinely recommended for patients with advanced/metastatic ovarian cancer with homologous DNA repair deficiency following standard chemotherapy. The FDA has now approved four different PARP inhibitors for clinical use: Olaparib, Rucaparib, Niraparib and Talazoparib (Huang *et al*, 2020). Synthetic lethality screens are designed to be high‐throughput and thus offer the possibility of discovering multiple synthetic lethal pairs that may be relevant in different cancer types, though as we will discuss, they have a number of limitations.

### RNAi and CRISPR screens

The first synthetic lethal screens were conducted using RNA interference technology. With this approach, short hairpin RNA (shRNA) and short interfering RNA (siRNA) sequences are designed to contain a short “seed sequence” that can bind to and downregulate mRNA to repress expression of the gene of interest (Sims *et al*, 2011). However, it was found that shRNA and siRNA could bind to and cause the downregulation of mRNAs unrelated to the gene of interest, so‐called “off‐target effects”, that resulted in false‐positive hits in functional genomic screens. In response, there have been informatics tools developed to eliminate false‐positive data from off‐target effects (Tsherniak *et al*, 2017). Recently developed CRISPR technology has proven to be much more specific, offering an efficient and robust tool for high‐throughput screening. Generally, CRISPR screens work by using custom‐designed guide RNAs (gRNAs) with a target sequence of 20 basepairs to direct Cas9 to sequence‐specific regions of the genome, in order to induce a double‐strand DNA break thus resulting in precise biallelic loss‐of‐function mutations (Basheer & Vassiliou, 2019). Variations of this technology have also been developed, including CRISPR interference (CRISPRi) that results in suppression of gene expression rather than excision of genomic DNA sequences, base editing, which allows for specific base pair modifications, and RNA targeting, where enzymes recognize and edit mRNA sequences rather than DNA (Huang *et al*, 2020). There is also a CRISPR strategy designed to target fusion oncogenes through targeting of two intronic sequences, one from each gene in the fusion oncogene, without affecting the genes in germline non‐rearranged alleles (Martinez‐Lage *et al*, 2020). Combinatorial CRISPR screens are increasingly being used to identify synthetic lethal targets (Thompson *et al*, 2021). Zhou and colleagues demonstrate the efficient knockout of three genes simultaneously in a human ovarian cancer cell line, to illustrate their technology can be applied to screen for synergistic anti‐cancer genetic combinations (Zhou *et al*, 2020). Gier and colleagues developed a similar approach, but using a Cas12a‐based system, to perform a double‐knockout screen in murine leukaemia cells to uncover synthetic lethal interactions (Gier *et al*, 2020). How efficient these vector systems can be translated to PDTOs and PDTX models has not yet been explored, but these approaches offer promise in identifying synthetic lethal targets. Notably, it has recently been demonstrated that chromothripsis, a single event that results in extensive clustered chromosomal rearrangements, occurs as an on‐target consequence of CRISPR‐Cas9 genome editing. Therefore, such events also need to be considered and carefully monitored in experimental models that incorporate this approach (Leibowitz *et al*, 2021).

### High‐throughput functional genomic screens using human cancer cell lines

Project DRIVE, from Novartis (McDonald *et al*, 2017), and Project Achilles, from the Broad Institute (Cowley *et al*, 2014), both used a library of shRNA to screen hundreds of human cancer cell lines from the Cancer Cell Line Encyclopedia (CCLE) project. Both projects described oncogenic addictions whereby cell lines driven by common oncogenes, such as KRAS, NRAS and BRAF, exhibited increased dependency and thus sensitivity to suppression by shRNA. As expected, KRAS mutation dependence was observed in colon, pancreatic and lung cancer cell lines, NRAS mutation dependence in melanoma lines, and BRAF mutation dependence in colon, thyroid and melanoma lines (McDonald *et al*, 2017). The presence of certain oncogenes also predicted dependency on other genes, such as with PIK3CA mutated cell lines demonstrating a preferential dependency on MTOR (Cowley *et al*, 2014). These projects also described examples of known collateral synthetic lethality which are synthetic lethal relationships among paralog genes for which dependency on one paralog is conferred by loss of a second functionally redundant paralog gene. Examples of this include SMARCA2/SMARCA4, ARID1A/ARID1B, UBB‐UBC and VPS4A/VPS4B. The latter pair, VPS4A/VPS4B, was further examined in a report by Neggers and colleagues who identified this pair using CRISPR‐spCas9 and RNAi loss‐of‐function screens. They found that VPS4A is essential in cancers with VPS4B loss adjacent to SMAD4 on chromosome 18q, and VPS4B is required in tumours with co‐deletion of VPS4A and CDH1 (E‐cadherin) on chromosome 16q (Fig 2). They performed *in vivo* functional validation of this synthetic lethal relationship by inducing CRISPR‐SpCas9‐mediated VPS4A suppression in rhabdomyosarcoma and pancreatic ductal adenocarcinoma PDTXs deficient of VPS4B and showed this resulted in near‐complete tumour regression (Neggers *et al*, 2020). Therefore, loss of 18q (VPS4B and SMAD4) or loss of 16q (VPS4A and CDH1) are potential predictive biomarkers for response to therapies directed at the synthetic lethal pair, VPS4A or VPS4B, respectively. Unfortunately, no selective VPS4 inhibitors are currently available, but this represents a promising area for drug development (Szymańska *et al*, 2020).

A recent effort by Behan and colleagues from the Sanger Institute used CRISPR/Cas9 screens on 324 human cancer cell lines from 30 different types of cancer. They identified good therapeutic targets as fitness genes restricted to specific molecular contexts or histologies and identified 617 cancer type‐specific and 92 pan‐cancer targets. This work demonstrated that a context‐dependent and histology‐dependent approach can result in the discovery of more novel therapeutic targets (Huang *et al*, 2020). Among the signals identified was Werner syndrome ATP‐dependent helicase (WRN), a synthetic lethal target in tumours with microsatellite instability, which was also discovered in previous large‐scale RNAi and CRISPR screens (Chan *et al*, 2019). CRISPR‐mediated knockout of WRN in microsatellite unstable cell lines from colon, ovarian, endometrial and gastric cancers decreased their fitness compared to wildtype, offering a novel therapeutic target in these rare subsets of cancers (Behan *et al*, 2019; Chan *et al*, 2019).

Han and colleagues established a high‐throughput method to propagate ~200 million cells simultaneously in 3D spheroid culture to perform genome‐wide CRISPR screens using a single guide RNA (sgRNA) library on H23, a human lung adenocarcinoma cell line (Han *et al*, 2020). They identified carboxypeptidase D (CPD) as a top synthetic lethal hit in H23 cells (which harbour a KRAS‐G12C mutation) treated with a KRAS inhibitor, ARS‐853. Takahashi and colleagues demonstrated that NRF2 was required for NSCLC cell lines to generate 3D spheroids, and using a CRISPR‐Cas9 screen, showed NSCLC cells with NRF2 upregulation had a high dependency on GPX4. This was functionally validated by using GPX4 inhibitors, ML210 and RSL3, on 3D spheroid culture, which resulted in decreased cell survival in 3D spheroid culture (Takahashi *et al*, 2020). In these instances, a KRAS‐G12C mutation or NRF2 upregulation represent potential predictive biomarkers for CPD and GPX4 inhibition, respectively.

### In vivo functional genomic screens

RNAi and CRISPR screens have also been applied to *in vivo* xenograft models. Using an shRNA library, an *in vivo* xenograft model established from a human colon cancer cell line showed that knockdown of TMED2 and SOX12 enhanced metastatic spread of colon cancer cells (Duquet *et al*, 2014), and an *in vivo* xenograft model established from human pancreatic ductal adenocarcinoma cells showed that knockdown of WD repeat‐containing protein 5 (WDR5), a member of the COMPASS histone H3K4 methyltransferase complex, resulted in dramatic tumour growth arrest (Carugo *et al*, 2016). In another example, Wei and colleagues used a targeted CRISPR screening approach on a PDTX model established from poorly differentiated metastatic pancreatic ductal adenocarcinoma, known to contain KRAS, p53 and SMAD4 mutations. They identified PRMT5 as a therapeutic target for cells co‐treated with Gemcitabine chemotherapy (Wei *et al*, 2020).

Current approaches for using RNAi and CRISPR functional genomic screens on *in vivo* xenograft models necessitates the use of human cancer cell lines that can be cultured for a period of time *in vitro* to allow for antibiotic selection of successfully transduced cells that are then transplanted into mice. A modified CRISPR vector developed by Hulton and colleagues, pSpCTRE, is able to reduce the time required for *in vitro* culture. This vector encodes CD4 as a compact cell surface selection marker, a tetracycline response element (TRE)‐promoter that is engineered to minimize leaky Cas9 expression for tight temporal control of Cas9 activity critical for avoiding aberrant genome‐editing activity, and the reverse tetracycline‐controlled transactivator (rtTA)‐V10 variant that displays increased sensitivity to low doxycycline concentrations in order to improve Cas9 expression in response to doxycycline treatment *in vivo* (Hulton *et al*, 2020). This approach holds promise for allowing CRISPR screens to be conducted on PDTXs.

### Genotype–phenotype relationship mapping

Advancements in sequencing technology have spurred the genomic revolution allowing for high‐quality deep sequencing of the human genome and sequencing of thousands of human tumours. However, in order to interpret this information, genotype–phenotype relationships need to also be defined. Reverse genetic screens aim to introduce gene disruptions in order to analyse the resultant phenotype, whereas forward genetic screens seek to identify the gene(s) responsible for a particular phenotype. These studies represent the majority of experimentally validated information on genotype–phenotype relationships. However, it is becoming readily apparent that genotype–phenotype relationships are exceedingly complex, and not limited to a single gene corresponding to a single phenotype, where certain genetic mutations do not consistently result in the same phenotype, due to factors such as incomplete penetrance, variable expressivity, and the multitude of interactions a single gene product can have (Vidal *et al*, 2011). Alternative pre‐mRNA splicing due to cis‐acting regulatory sequences, and trans‐acting splicing factors, has also been shown to affect genotype–phenotype relationships (Baeza‐Centurion *et al*, 2019).

One approach for defining genotype–phenotype relationships in cancer has been to map “interactome” networks using a computational systems biology approach in conjunction with high‐quality and extensive genomic, transcriptomic and proteomic data to map macromolecular interactions, including protein–protein interactions, protein‐nucleic acid interactions, post‐translational modifications and their targets. An approach taken by Rolland and colleagues resulted in a systematic map of ~14,000 high‐quality human binary protein–protein interactions and found that cancer‐associated proteins tend to form subnetworks that are highly connected and represent different processes required for tumorigenesis (Rolland *et al*, 2014). This was later expanded with the integration of genomic, transcriptomic and proteomic data to identify 53,000 protein–protein interactions, serving as a genotype–phenotype reference map (Luck *et al*, 2020).

## Pharmacogenomics and drug screening platforms

### Pharmacogenomics studies using human cancer cell lines

The field of cancer pharmacogenomics aims to combine information about cancer genomics with knowledge from the pharmacology of anti‐cancer compounds in order to predict which treatments will be the most effective. Drug screening of hundreds of chemical compounds against human cancer cell lines have been made publicly available in databases such as the Cancer Therapeutics Response Portal (CTRP; Seashore‐Ludlow *et al*, 2015) and the Genomics of Drug Sensitivity in Cancer (GDSC; Iorio *et al*, 2016).

Iorio and colleagues from the GDSC project analysed cancer‐driven genetic alterations in 11,289 tumours from 29 different tissues. They defined “cancer functional events” as a mutation detected from whole‐exome sequencing that was consistent with positive selection, a focal recurrently aberrant copy number segment or hypermethylated promoter that could be mapped to the 1,001 human cancer cell lines annotated with molecular information. They tested on these lines 265 compounds that were approved for clinical use, under development, or purely experimental, and found 688 statistically significant associations between the cancer functional event and compounds tested (Iorio *et al*, 2016). One interesting association discovered was a sensitivity of squamous lung cancer cell lines harbouring inactivating mutations of MLL2 (a chromatin modifier) to the anti‐androgen compound Bicalutamide. Another association was sensitivity of gastric cancer cell lines with truncating mutations of BCOR (a transcriptional corepressor) to the protein kinase Cß inhibitor, LY317615 (Huang & Vakoc, 2016). The mechanism for these novel drug‐mutation associations has not been established and certainly requires further pre‐clinical validation.

It is encouraging that the pharmacogenomics relationships found in the study by Iorio and colleagues were validated when comparing to prior studies from the Cancer cell Line Encyclopedia (CCLE; Barretina *et al*, 2012; Seashore‐Ludlow *et al*, 2015). In two studies published in 2012, the Cancer Genome Project (CGP; Garnett *et al*, 2012), and the CCLE (Barretina *et al*, 2012), there was the suggestion of discordant results attributed to a lack of standardization of experimental procedures and analysis methods (Haibe‐Kains *et al*, 2013). However, further analyses of these datasets found that the inconsistent number of cell lines sensitive to drug therapies was due to differences in experimental and analytical procedures (Cancer Cell Line Encyclopedia Consortium & Genomics of Drug Sensitivity in Cancer Consortium, 2015) and that there was a reasonable agreement in drug sensitivity between the two studies (Bouhaddou *et al*, 2016; Geeleher *et al*, 2016; Mpindi *et al*, 2016). In addition, there appears to be a high degree of concordance between two large datasets that examine genetic dependencies in human cancers using CRISPR screens (Dempster *et al*, 2019; Pacini *et al*, 2021), despite different experimental procedures, which provides confidence in the reproducibility of such large‐scale studies.

A novel approach to drug screening called PRISM (profiling relative inhibition simultaneously in mixtures) was developed by Corsello and colleagues. This approach increases the number of drug‐cell line combinations that can be tested simultaneously by labelling cancer cell lines each with a unique DNA sequence (or “barcode”), pooling the cell lines in equal proportions, and then using the barcodes (detected by DNA sequencing) as a surrogate readout for cellular viability after a period of *in vitro* drug exposure. They used this to test 4,518 drugs against 578 cell lines spanning 24 different tumour types (Corsello *et al*, 2020).

Large‐scale pharmacogenomics studies are helpful to identify potential novel treatments for specific genetic alterations but must be followed by further validation studies. Standardization of *in vitro* drug screening and analysis methods will also help to reconcile the interpretation of drug sensitivities and genetic dependencies identified from these studies.

### PDTOs, PDTXs, and other models for drug screening

PDTOs have recently gained favour for testing of drug compounds given they can be maintained *in vitro*, are amenable to high‐throughput analyses and are thought to be more representative of the cellular and mutational heterogeneity of the original patient’s disease. PDTOs have been used to test drug sensitivity in almost all tumour types, including colon (van de Wetering *et al*, 2015; Verissimo *et al*, 2016), breast (Sachs *et al*, 2018), lung (Kim *et al*, 2019), prostate (Gao *et al*, 2014; Yan *et al*, 2018, 2019) and pancreas cancers (Huang *et al*, 2015; Schuster *et al*, 2020).

Schuster and colleagues developed a microfluidics platform that allows for dynamic and combinatorial drug screening on PDTOs. They used this platform to demonstrate that some patient‐derived pancreatic ductal adenocarcinoma organoids were more sensitive to temporally pulsed treatment with 5‐fluorouracil and gemcitabine‐based chemotherapy regimens, compared to others for which a constant presence of chemotherapy resulted in greater response (Schuster *et al*, 2020). This illustrates the power of *in vitro* organoid cultures systems that allow for the simultaneous screening and real‐time monitoring of PDTOs to different treatment regimens in order to determine the ideal treatment for a given patient’s cancer.

For many years, PDTXs have been used to test tumour sensitivity to drug therapy *in vivo* in various cancer types (Invrea *et al*, 2020), as these models most closely mimic the genetic heterogeneity and cellular architecture of the original tumour. For example, Gao and colleagues established over 1,000 PDTXs on which they tested 62 treatments and were able to identify genotype‐drug sensitivity associations, and mechanisms of drug resistance (Gao *et al*, 2015). PDTX models have also been used recently to study the effects of novel cell‐based therapies, such as CAR‐T‐cell therapy (Teng *et al*, 2019), and the effects of treatments designed to target cancer stem cells (Tosoni *et al*, 2017).

An interesting approach to simultaneously test PDTX response to multiple chemotherapeutic agents *in vivo* is to use liposomal nanoparticles, each containing a different agent as well as a double‐stranded DNA barcode sequence that is unique and can be used to decode which cell received which chemotherapeutic agent. Yaari and colleagues used this approach to deliver different treatments (Doxorubicin, Cisplatin or Gemcitabine) to PDTXs established from triple‐negative breast cancer and used the barcodes (quantified from DNA sequencing) to decode which cells received which chemotherapeutic agent, and the efficacy of each treatment (Yaari *et al*, 2016). However, the limitation of such an approach is that one tumour cell can theoretically be treated with more than one chemotherapeutic agent, and further, that the tumour microenvironment can influence treatment sensitivity, an effect that is confounded when the microenvironment contains cells treated differently.

Methods have also been developed to test drug sensitivity on patient tumours without the use of cancer models. One such approach uses a microfluidics platform to simultaneously test different combination therapies on biopsy material *ex vivo*, eliminating the need for *ex vivo* tissue culture (Eduati *et al*, 2018). The limitation with this approach is that the primary tumour may contain a mutational heterogeneity that is distinct from sites of metastatic disease, and predictions derived from drug sensitivity testing in the primary tumour will then not predict the response of distant metastases.

## Future directions in pre‐clinical drug screening and biomarker discovery

The multitude of functional genomic screens and pharmacogenomics studies described in this review have led to the identification of several novel therapeutic targets, some of which do not yet have targeted molecular therapies available and thus represent promising drug development opportunities. One lesson that is apparent from these studies is that the signal for a genetic dependency and/or therapeutic target is most likely to be disease context specific. While there has been significant work done to identify genetic dependencies from large‐scale studies from the Wellcome Sanger Institute and the Broad Institute as part of the Cancer Dependency Map, there is a call for additional research groups to collaborate on this international initiative in order to evaluate all possible gene–drug perturbation combinations in all cancer types (Boehm *et al*, 2021). While ambitious, this initiative will hopefully identify novel drug therapy options in subsets of human cancer with specific genetic or epigenetic alterations.

**Figure 3:**
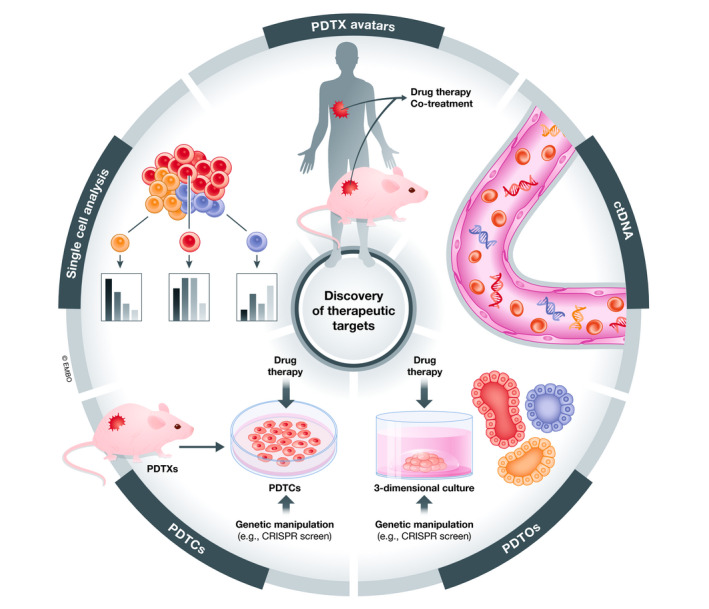
Approaches to discover novel therapeutic targets in cancer Depicted are approaches that hold promise for discovering novel therapeutic targets and biomarkers of disease response in human cancers. PDTX avatars are co‐treated with the same therapies received by the patient, allowing for response to drug therapy and changes in clonal heterogeneity to be modelled *in vivo* (Invrea *et al*, 2020). Liquid biopsies to detect ctDNA represent a non‐invasive approach to evaluating tumour heterogeneity and response to therapy and may be helpful in studying the effect of drug therapy in patients in a clinical trial setting (Wan *et al*, 2017). PDTOs preserve spatial architecture *in vitro* (Clevers, 2016), and PDTCs are short‐term cultures derived from PDTX cells (Bruna *et al*, 2016b), both of which can be used to evaluate response to drug therapy and genetic manipulation. Single‐cell analysis of the genome, transcriptome, proteome and epigenome may offer layers of information for understanding response of tumour cells to drug therapy (Georgopoulou *et al*, 2021), an advantage over current drug therapy screens that mainly assess only cell viability.

The application of single‐cell analysis may also offer useful information that can improve the identification of novel therapeutic targets. Single‐cell analysis of genomic DNA can reveal the diversity of genomic clones present which otherwise would have been analysed in bulk, whereas single‐cell RNA sequencing offers the opportunity to assess the heterogeneity in transcriptomic profiles of cancer cells in response to treatment (Tognetti *et al*, 2021). Single‐cell protein analysis is also possible using CyTOF, although only a limited number of markers can be tested at the time (Sahaf *et al*, 2020). Recently, CyTOF analysis of PDTXs and PDTCs has been combined with drug screening to identify cell phenotype biomarkers to predict drug sensitivity or resistance (Georgopoulou *et al*, 2021). As advanced single‐cell technologies become more accessible, it will be more feasible to incorporate them into large therapeutic screens allowing for high‐content information such as changes in gene expression to drug treatment rather than relatively simple measures, such as cellular viability and proliferation (Fig 3). This added information has the potential to identify patterns of genetic and/or epigenetic dependencies that may represent novel therapeutic targets alone or in combination with other therapies.

In the era of personalized medicine, the approach of using patient “avatars” has come into favour (Fig 3). This approach involves establishing xenografts from patient tumours, at the stage of initial biopsy or surgical resection, and to treat the established lines similarly to how the patient is treated, in order to predict treatment response and/or development of treatment resistance in real‐time, with the aim of this information being used to guide future clinical decision‐making (Invrea *et al*, 2020). The main limitation of this approach is the extensive resources required to establish and maintain the PDTXs. The time required to establish these avatars, and assess treatment response may exceed the window in which this information would be helpful for clinical decision‐making. Nevertheless, PDTXs most closely mimic a patient’s disease and will continue to be the gold standard for pre‐clinical drug testing. Indeed, if enough PDTX models are generated to represent inter‐tumour heterogeneity, testing of models that are representative of a given patient tumour, although not individually matched, will represent a significant advance. The development of cancer organoid models that can be maintained *in vitro* in 3‐dimensional culture may prove to be useful for functional genomic screens to identify novel therapeutic targets and for validation of genetic dependencies in a highly controlled system (Fig 3). Adaptations of such PDTX and PDTO models also allow for elements of the tumour microenvironment, including interaction with the immune system to be studied, which also represents a substantial area of progress in improved drug development.

## Conclusions

The discovery of novel therapeutic targets and predictive biomarkers that can guide effective anti‐cancer therapies have the potential to significantly improve patient survival. Progress in this area of research will require further characterization of human cancer models, and their adaptation for use with functional genomic screens to identify genetic dependencies in cancers. Single‐cell analysis approaches have the potential to reveal the heterogeneity of response from cancer cells to drug therapy and to help uncover mechanisms of drug resistance that need to be overcome. Over the past few years, several novel therapeutic targets have been identified using these approaches, and with further validation studies, hold promise for clinical implementation. The identification and development of additional therapeutic targets and predictive biomarkers, however, is likely to require an approach that considers disease‐specific contexts and the integration of multi‐component high‐content drug‐response information.

## Conflict of interest

C.C. is a member of AstraZeneca’s iMED External Science Panel, of Illumina’s Scientific Advisory Board, and is a recipient of research grants (administered by the University of Cambridge) from AstraZeneca and Varsity Pharmaceuticals. The other author declares no conflict of interest.

## For more information

i An encyclopaedia of breast cancer patient‐derived tumour xenografts (https://caldaslab.cruk.cam.ac.uk/bcape/).
ii COSMIC, a catalogue of somatic mutations in human cancer (https://cancer.sanger.ac.uk/cosmic).
iii Cellosaurus, a resource of information on cell lines from multiple species, not limited to human cancer (https://web.expasy.org/cellosaurus/).
iv Cell Model Passports, which provides genomics information and functional datasets on human cancer cell lines and patient‐derived tumour organoid models (https://cellmodelpassports.sanger.ac.uk/).
v Cancer Therapeutics Response Portal (CTRP) from the Broad Institute that links the genetic information from human cancer cell lines with their response to small‐molecular therapies (https://portals.broadinstitute.org/ctrp/).
vi Cancer Dependency Map (DepMap), an initiative from the Broad Institute and Wellcome Sanger Institute that aims to map all the genetic dependencies of human cancers (https://depmap.org/portal/).
vii Genomics of Drug Sensitivity in Cancer (GDSC), collaboration between The Cancer Genome Project at the Wellcome Sanger Institute and the Center for Molecular Therapeutics to discover therapeutic biomarkers in human cancers (https://www.cancerrxgene.org/).
viii MD Anderson Cell Lines Project (MCLP) provides protein expression data linked to genomic and transcriptomic data in human cancer cell lines (https://bioinformatics.mdanderson.org/public‐software/mclp/).

**Pending issues**

- Determine whether ctDNA can be used to accurately assess drug response and changes in clonal heterogeneity of a patient’s tumour.
- Determine whether drug response and changes in clonal heterogeneity in PDTO and PDTX models of different human cancers correspond to that seen in similarly treated patients.
- Incorporate single‐cell genomic, transcriptomic, proteomic and epigenomic analysis into drug therapy screens in order to identify novel predictive biomarkers and therapeutic targets in human cancers.
